# *Mycobacterium tuberculosis*-Induced Maternal Immune Activation Promotes Autism-Like Phenotype in Infected Mice Offspring

**DOI:** 10.3390/ijerph18094513

**Published:** 2021-04-23

**Authors:** Wadzanai Manjeese, Nontobeko E. Mvubu, Adrie J. C. Steyn, Thabisile Mpofana

**Affiliations:** 1 Department of Human Physiology, School of Laboratory Medicine and Medical Sciences, College of Health Sciences, University of KwaZulu Natal, Durban 4001, South Africa; wadzymanjeese@gmail.com; 2 Discipline of Microbiology, School of Life Sciences, College of Agriculture, Engineering and Science, University of KwaZulu Natal, Durban 4001, South Africa; mvubuN@ukzn.ac.za (N.E.M.); adrie.steyn@ahri.org (A.J.C.S.); 3 Africa Health Research Institute, K-Rith Tower Building, Nelson Mandela School of Medicine, Durban 4001, South Africa; 4 Department of Microbiology, University of Alabama, Birmingham, AL 35294, USA

**Keywords:** maternal immune activation, *Mycobacterium tuberculosis*, cytokines, social behaviors, Autism, synaptic genes, restrictive repetitive patterns

## Abstract

The maternal system’s exposure to pathogens during pregnancy influences fetal brain development causing a persistent inflammation characterized by elevated pro-inflammatory cytokine levels in offspring. *Mycobacterium tuberculosis* (*Mtb*) is a global pathogen that causes tuberculosis, a pandemic responsible for health and economic burdens. Although it is known that maternal infections increase the risk of autism spectrum disorder (ASD), it is not known whether *Mtb* infection is sufficient to induce ASD associated behaviors, immune dysregulation and altered expression of synaptic regulatory genes. The current study infected pregnant Balb/c mice with *Mtb* H37Rv and valproic acid (VPA) individually and in combination. Plasma cytokine profiles were measured in offspring using the Bio-plex Th17 pro mouse cytokine panel. *Mtb* infection increased plasma interleukin (IL)-6 and IL-17A, while tumor necrosis factor alpha (TNF-α), interferon (IFN)-γ and IL-1β were reduced when compared with saline. *Mtb*-induced maternal immune activation (MIA) offspring displayed increased grooming behavior. The study also revealed dysregulation in gene expression of synaptic molecules in the cerebellum. MIA rescued the VPA-induced effects on self-grooming and social interaction behaviors. Our finding therefore highlights a potential role of *Mtb* as a MIA agent that can potentially contribute to ASD.

## 1. Introduction

Autism spectrum disorder (ASD) is a group of neurodevelopmental disorders (NDDs) caused by a complex interaction between genes and prenatal environmental factors. It is characterized by repetitive behaviors, communication deficits and lack of social interaction skills [1]. Maternal immune activation (MIA) caused by infections in pregnancy are an important environmental risk factor that can influence the developing brain. Second trimester respiratory infections and bacterial infections increase the risk of NDDs such as ASD [2,3]. The maternal system strikes a balance between immune tolerance of the growing fetus and immune response against invading pathogens [4]. Maternal immune response to infection triggers production of inflammatory cytokines which can traverse the fetal–placental barrier inducing fetal inflammation that disrupts brain development. This inflammation persists through postnatal life as evidenced by elevated pro-inflammatory cytokines in the CNS and serum/plasma of ASD patients [5,6]. Maternal infections of Poly I:C, LPS, influenza, *C. rectus* and *L. monocytogenes* alter cytokine levels in the placenta, amniotic fluid, fetal brain and plasma [4,7,8,9,10]. Cytokines mediate neuro-immune communication, and they also regulate neurogenesis and synaptic plasticity; hence, an imbalance in cytokine levels during development can be detrimental to the developing brain.

Several genes have been implicated in ASD and they are known to converge on biological pathways that are involved in the regulation of neuronal activity [11,12]. These genes include SH3 and multiple ankyrin repeat domains 3 (*SHANK3*), neurexins (*NRXN*s) and neuroligins (*NLGNs*) which encode proteins involved in synaptic transmission and plasticity [13]. Neurexins are pre-synaptic cell adhesion molecules encoded by *NRXN*1, 2 and 3 genes, while *NLGN*s are post-synaptic cell adhesion molecules encoded by *NLGN*1, 2, 3 and 4 [13,14]. *SHANK3*, *NRXN1*, *NRXN2*, NLGN1 and *NLGN2* are candidate NDD susceptibility genes. Mutations and deletions in these genes are implicated in the pathophysiology of ASD [15,16,17]. *NLGN1* and *NLGN2* are expressed on excitatory and inhibitory synapses, respectively, while *NLGN3* and 4 are found in both synapses. Changes in expression patterns of *NRXNs* and *NLGNs* affect the synapse excitation/inhibitory (E/I) balance leading to altered information processing.

Valproic acid (VPA) is an anticonvulsant and mood stabilizer that is used to treat epilepsy and depression disorders [18]. Treating pregnant women with VPA is associated with higher incidence of ASD and physical deformities in children [19,20]. It has been extensively shown that gestational VPA exposure of mice on E12.5 induces ASD-traits in offspring, which include social deficits, repetitive behaviors, communication challenges and neuronal damage [21,22,23,24]. The VPA model is a well-established tool for studying ASD research, owing to its ability to recapitulate human symptoms of ASD.

In this study we used *Mycobacterium tuberculosis (Mtb)* H37Rv to induce MIA in pregnant mice. *Mtb* is a global pathogen that is common in pregnant women, and children born to them are at an increased risk of having low birth weight, infant mortality and prematurity [25,26]. *Mtb* causes tuberculosis, a highly prevalent disease in Africa that causes social and economic challenges. Conversely, neurodevelopmental disorders are becoming increasingly common even though they are poorly understood and underdiagnosed in Africa [27]. No study has evaluated maternal *Mtb* infection and its potential influence on ASD-associated genes and on the immune systems of infected mice’s offspring. This study evaluates *Mtb* infection and its possible link to ASD-like phenotype in the offspring of infected mice.

## 2. Materials and Methods

### 2.1. Animals

**Figure 1:**
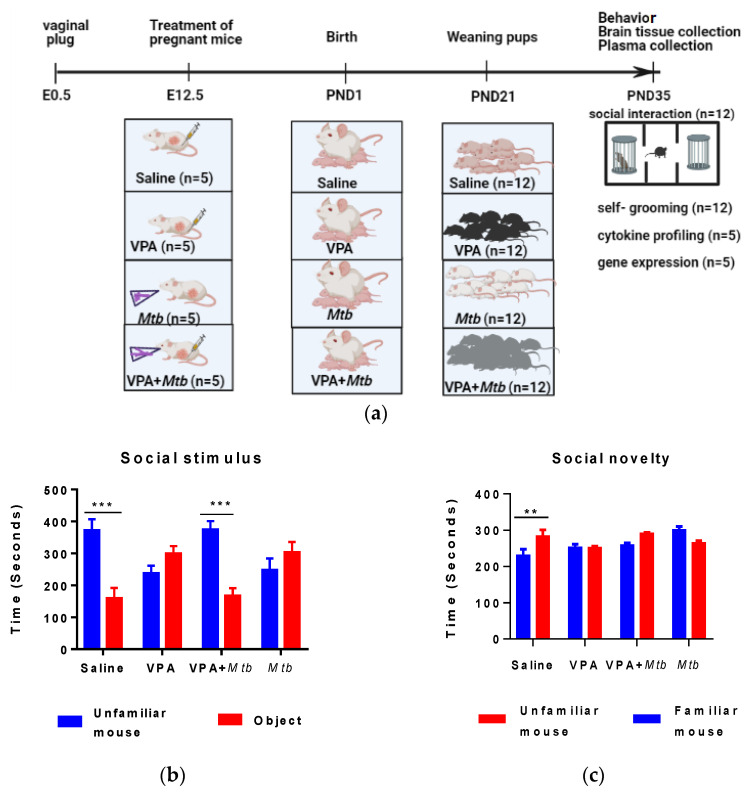

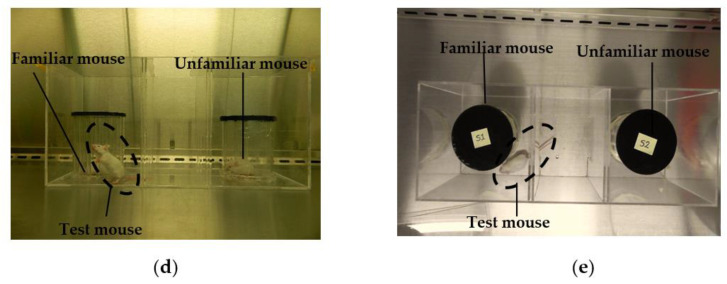
(**a**) Study design; (**b**) Social stimulus—Graph shows the average time spent with a familiar mouse compared with object. Unlike the saline offspring (*p* < 0.0001), *Mycobacterium tuberculosis (Mtb)* offspring spent more time with an object than a mouse. Valproic acid (VPA) + *Mtb* offspring spent significantly more time (*p* < 0.0001) with an unfamiliar mouse than the object. *** denotes *p*< 0.0001 (two-way ANOVA); (**c**) Social novelty—Graph shows the average time (±SEM) spent with an unfamiliar mouse compared with a familiar mouse in a 10 min phase. Unlike the saline offspring (*p* < 0.001), *Mtb* offspring failed to choose the unfamiliar mouse, while *VPA + Mtb* offspring chose the unfamiliar mouse. ** denotes *p* < 0.001 (*n* = 12 per group, two-way ANOVA); detailed statistics information is available in Appendix B, Table A3 and Table A4; (**d**) Representative images of the side-view and (**e**) aerial view of social novelty phase during the three-chamber social interaction test.

Balb/c mice at 8 weeks of age were obtained from Africa Health Research Institute (AHRI), South Africa. The study followed the Animal Research Reporting of In Vivo Experiments (ARRIVE) guidelines, and animals were handled according to the principles of National Institutes of Animal Care and Use of Laboratory Animals of the National Academy of Sciences. Females were group-housed (5 per group) for 5 days to synchronize their estrous cycle. Bedding from the male cages was added to the female cages to induce the estrous phase. The mice were mated on a ratio of 1:1 (male to female) overnight. Vaginal plugs were checked early morning, males were immediately removed upon confirmation of vaginal plug presence and this confirmation marked embryonic day 0.5 (E0.5). In our pilot study, administration of 500 mg/kg of VPA and *Mtb* together induced complete fetal resorption; hence, for this study we lowered the VPA dose to 350 mg/kg to increase fetal survival rate in the VPA + *Mtb* group. The VPA group was the positive control of the study, and the VPA + *Mtb* group was created to assess the effects of dual exposure to two ‘’environmental insults’’. Twenty pregnant mice were randomly assigned to 4 groups (5 per group), and the treatments were administered on E12.5, as shown in Table 1 and Figure 1a. Only 3 animals per group had successful pregnancies; hence, litters were culled to 12 per group and no more than 4 animals per litter (2 per sex). All animals were raised in a biosafety level 3 laboratory (BSL3). Each animal raised her litter under 12 h light/dark cycle and a constant supply of food and water. Litters were weaned at postnatal day (PND) 21, at which time males and females were separated but grouped according to treatments. Five animals were randomly picked from all the litters for cytokine and gene expression profiles.

### 2.2. Mtb Infection

Ten pregnant mice from the *Mtb* and VPA + *Mtb* groups were placed in a Glas-col infection chamber (Terre Haute, IN, USA) with 10 mL of 1 × 10^8^ CFU (OD_600_ = 1) *Mtb* H37Rv inoculum expelled into the nebulizer using a syringe. The Glas-col machine cycle was set to the following conditions: preheat (15 min), nebulization (30 min), cloud decay (30 min), decontamination (15 min), cool down (10 min). The mice were carefully removed from the machine using forceps and placed into cages with bedding, food and water.

### 2.3. Social Interaction Test

This test was performed on PND 35 as previously described [28]. All procedures were conducted in a biosafety level 3 cabinet. Briefly, a 40 × 15 cm transparent Perspex box divided in three compartments with the following measurements: sides (15 × 15 cm); central (10 × 15 cm). The central compartment was connected to the side compartments by 7.5 cm sliding doors. A camera (Microsoft Lifecam Studio, Beijing, China) was placed above the apparatus to record all the areas of the three-chamber box during the test. The test was conducted in 3 phases: (i) Habituation phase—each mouse was allowed acclimatize and freely explore the chambers for 5 min; (ii) Social stimulus phase—an unfamiliar sex and age matched mouse was placed in a cylinder in 1 of the side chambers; the other side chamber contained an identical cylinder that was empty; and (iii) Social novelty phase—A novel sex- and age-matched mouse was placed in a cylinder in 1 of the side chambers while the other side chamber housed the familiar mouse that was encountered during the social stimulus phase (Figure 1d,e). During each phase, the test animal was placed in the center compartment and doors were opened to test the animal’s preferred compartment. The total amount of time spent in each compartment was analyzed and scored using BORIS software.

### 2.4. Self-Grooming/Repetitive Behaviors

To assess behaviors in the repetitive domain, the total time spent grooming (scratching and licking) was observed during the habituation phase of the three-chamber social interaction test. Five-minute videos were recorded, and the cumulative time spent grooming during the sessions were later scored by an investigator blinded to the treatments using Behavioral Observation Research Interactive Software (BORIS, v 7.7.4. Turin, Italy).

### 2.5. Blood Collection

On PND 35, balb/c mice were humanely sacrificed using halothane overdose; thereafter, cardiac puncture was performed to collect blood into EDTA-coated 1 mL tubes. The tubes were left standing for 30 min before centrifuging at 10,000 rpm at room temperature for 10 min. Plasma samples were aliquoted and flash frozen on dry ice then stored at −80 °C until analyzed.

### 2.6. Brain Tissue Collection

On PND 35, five animals per group were slightly anaesthetized and decapitated using a guillotine. The brain tissue was carefully removed from the skull on ice, and the whole cerebellum was dissected and immediately frozen on dry ice then stored at −80 °C until analysis.

### 2.7. Relative mRNA Expression in the Cerebellum (Quantitative PCR)

Total mRNA was extracted from whole cerebellar tissues according to manufacturer’s instructions using the Zymo Research Quick-RNA Miniprep kit (Zymo Research, Irvine, CA, USA). Briefly, cerebellar tissues were mechanically homogenized in RNA lysis buffer, and the homogenate was centrifuged at 10,000× *g* for 30 s. The flow through was diluted (1:1) with ethanol and centrifuged on a Zymo-Spin IIICG column which was then treated with DNase 1 to digest genomic DNA. After recommended washing steps and centrifugation, RNA was eluted in 50 μL DNAse/RNAse free water. A nanodrop spectrophotometer (Thermo scientific Nanodrop 1000, Waltham, MA, USA) was used to assess the purity and concentration of the extracted RNA. A total of 1 μg of RNA was reverse transcribed into cDNA according to the manufacturer’s protocol using the iScript cDNA synthesis kit (Bio-Rad Laboratory Pty, Ltd., Hercules, CA, USA). All primers were designed using Primer-Blast designing program (http://www.ncbi.nlm.nih.gov/tools/primer-blast, accessed on 25 September 2019), then validated and optimized prior to the experiment. Real-time polymerase chain reaction (qPCR) conditions used were as follows: initial denaturation at 95 °C for 3 min followed by 40 cycles of polymerase activation and denaturation at 95 °C for 15 s, annealing/extension and plate read at 58 °C with a single fluorescent measurement. The housekeeping gene used in the analysis was glyceraldehyde-3-phosphate dehydrogenase (GAPDH) and the fluorescent dye utilized in the analysis was SYBR Green 1 (SSO Advanced Universal SYBR GR. SUPRMIX, Bio-Rad Laboratory, Hercules, CA, USA). The oligonucleotide primer sequences for the cerebellar synaptic genes investigated are shown in Table 2:

### 2.8. Cytokine Analysis

Cytokine analysis was conducted using a Bio-plex Pro™ Mouse Th17 Cytokine magnetic bead-based kit (Bio-Rad Laboratory, Hercules, CA, USA. Lot# 64313813). The kit was used to measure plasma levels of interleukin 6 (IL-6), interferon gamma (IFN-γ), interleukin 10 (IL-10), interleukin 1 beta (IL-1β), tumor necrosis factor alpha (TNF-α) and interleukin 17A (IL-17A). All steps were conducted according to the manufacturer’s instructions. Briefly, samples were diluted (1:4) in diluent provided by manufacturer. Antibody coupled beads were incubated with 50 μL of diluted plasma. After the recommended washes, biotinylated detection antibody was added to the beads and incubated. Streptavidin-PE was added to the beads, a Bioplex 200 System (Biorad) was used to measure fluorescent signals, and data were analyzed using Bio-plex Manager 5 Software. A 5-parameter model was used to calculate cytokine concentrations. Unknown concentrations were calculated based on a standard curve of known concentrations provided by the manufacturer. Cytokine concentrations below the limit of detection (LOD) were calculated as LOD/2 for statistics. Cytokine concentrations are expressed as pg/mL.

### 2.9. Statistical Analysis

Results were analyzed using GraphPad Prism software (version 7). The data were analyzed by one-way or two-way ANOVA followed by a Dunnett’s or Bonferroni multiple comparison post hoc test as appropriate to determine differences between the groups which were considered statistically significant when *p* < 0.05. Results are presented as the mean + standard error of the mean (mean + SEM) for *n* = 12 in behavioral tests and *n* = 5 for cytokine and gene expression analyses.

## 3. Results

### 3.1. The Effect of Prenatal Mtb Infection on Offspring Social Behaviors

The three-chamber social interaction test measured the sociability of animals in the social stimulus phase, where propensity to spend time with another mouse was compared with time spent with an object over a 10 min period. Although not statistically significant (*p* > 0.05), *Mtb* offspring spent more time with an object than a mouse (Figure 1b). Interestingly, mice born to VPA + *Mtb* treated mothers showed a preference for the unfamiliar mouse over the object (*p* > 0.0001), which indicated normal social behavior. We measured social novelty as the preference between a familiar mouse (previously encountered animal) and unfamiliar mouse. Mice are social animals that normally would show a preference for novel social experiences. In this phase, two-way ANOVA showed that there was no treatment effect on the choice between familiar and unfamiliar mouse; however, *Mtb* offspring were inclined to interact with the familiar mouse more than with the unfamiliar mouse (Figure 1c). Although not statistically significant (*p* > 0.05), mice born to VPA + *Mtb* treated mothers preferred the unfamiliar mouse to the familiar mouse.

### 3.2. Mtb Infection Increases Self-Grooming Behavior

**Figure 2:**
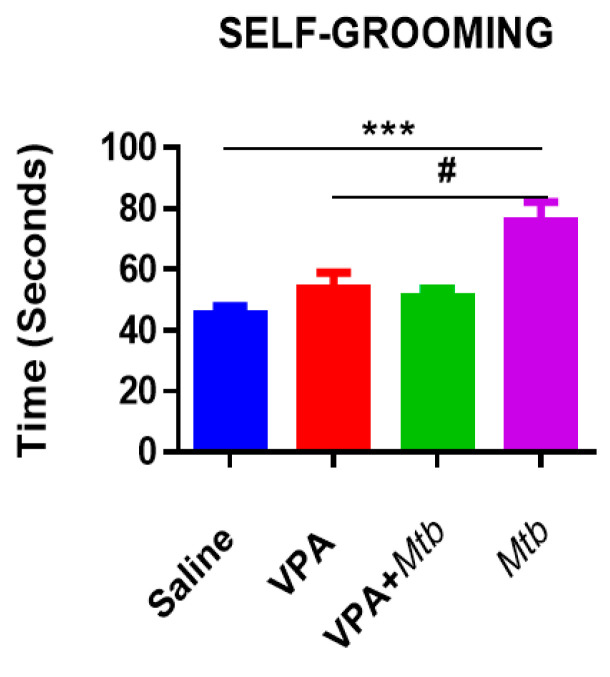
Graph showing total time spent while animals groomed themselves over 5 min (*n* = 12 per group). Prenatal exposure to *Mtb* significantly increased grooming when compared with saline and VPA, *p* < 0.0001 and *p* < 0.001, respectively. *** *p* < 0.0001 vs. saline and # *p* < 0.001 (one-way ANOVA). Detailed statistics information is available in Appendix B, Table A5.

The duration of whole-body grooming was monitored and recorded. An unusually long duration of a grooming pattern of the whole body was observed in this study. There was a significant increase (*p* < 0.0001 vs. saline; *p* < 0.001 vs. VPA) in grooming behaviors of the *Mtb* offspring (Figure 2). Interestingly, *Mtb* on its own was sufficient to increase self-grooming (*p* < 0.001 vs. VPA + *Mtb*) and dual exposure to VPA and *Mtb* did not increase the grooming behavior but restored it to the negative control (saline) frequency, suggesting a possible rescue mechanism of the grooming behavior.

### 3.3. Prenatal Mtb Infection Causes Immune Dysregulation in Offspring

**Figure 3:**
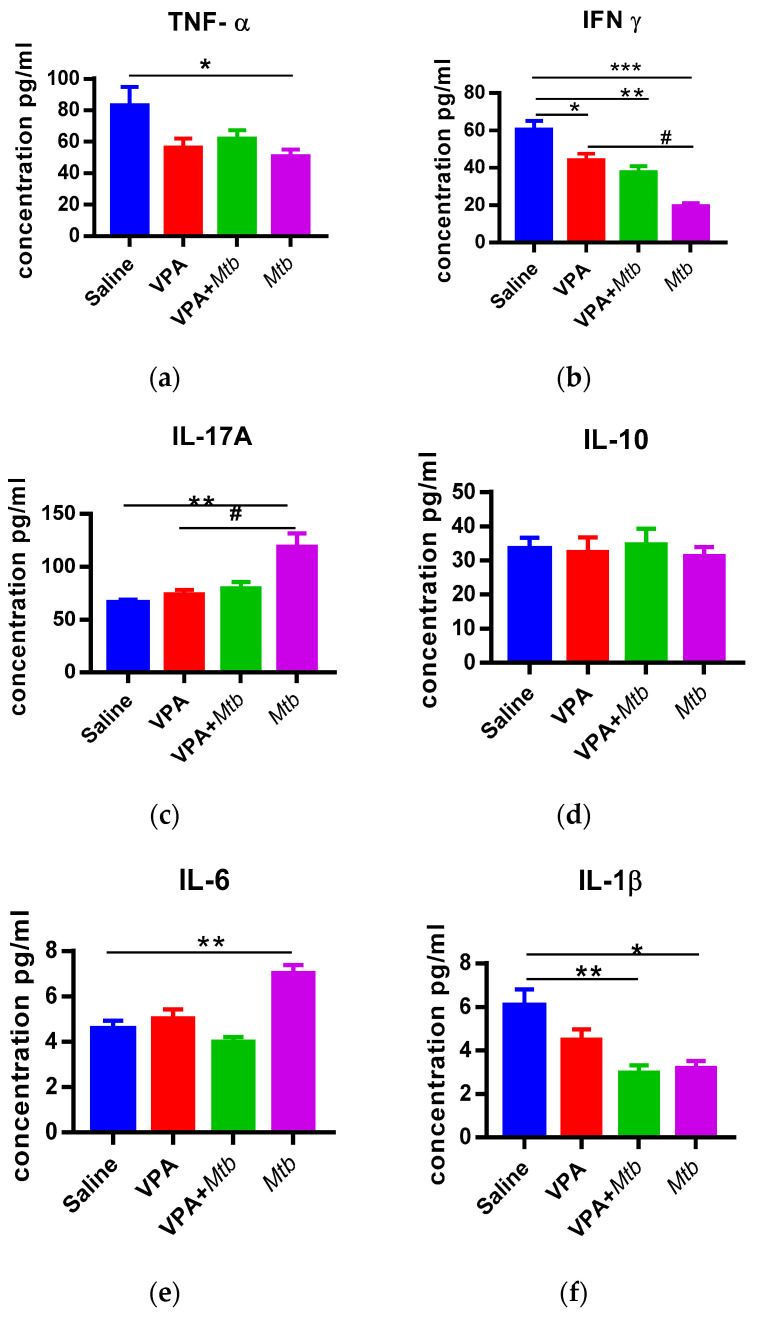
Graph showing concentrations of plasma cytokines at postnatal day (PND) 35 for (**a**) TNF-α (**b**) IFNγ (**c**) IL-17A (**d**) IL-10 (**e**) IL-6 (**f**) IL-1β. IL-6 and IL-17A plasma cytokine concentrations are heightened in *Mtb* offspring and restored to negative control levels in VPA + *Mtb* offspring. IL-1β, IFN-γ and TNF-α productions are reduced: # *p* < 0.001 vs. VPA, * *p* < 0.05, ** *p* < 0.001 and *** *p* < 0.0001 vs. saline; *n* = 5 animals per group (one-way ANOVA). Detailed statistics are available in Appendix B, Table A6.

The expression and subsequent production of IL-6, IL-1β, IFN-γ, IL-17A, TNF-α and IL-10 was measured in the plasma of MIA offspring. We found that IL-6 and IL-17A were significantly increased in mice that were prenatally exposed to *Mtb* infection (*p* < 0.001; *p* < 0.05 vs. saline respectively, Figure 3). Offspring from *Mtb* infected mice had significantly reduced TNF-α, IFN-γ and IL-1β (*p* < 0.05; *p* < 0.0001; *p* < 0.001, respectively) compared with saline exposed offspring. It was also observed that IFN-γ was reduced (*p* < 0.001) and IL-17A significantly elevated (*p* < 0.001) in the plasma of *Mtb* pups compared with VPA pups (positive control). *Mtb* infection had no effect on circulating IL-10 levels in the offspring. The VPA + *Mtb* offspring had significantly reduced plasma IFN-γ and IL-1β levels (*p* < 0.001) compared with saline offspring. *Mtb* on its own was sufficient to induce the elevation of IL-17A (*p* < 0.05) and IL-16 (*p* < 0.0001) compared with VPA and *Mtb* combined (VPA + *Mtb*) in the plasma of offspring (Appendix A). Taken together, there was a persistent dysregulation and imbalance in pro-inflammatory and anti-inflammatory cytokines in the plasma of MIA offspring.

### 3.4. Mtb Infection Dysregulates Gene Expression of Synaptic Molecules in Offspring Cerebellum

**Figure 4:**
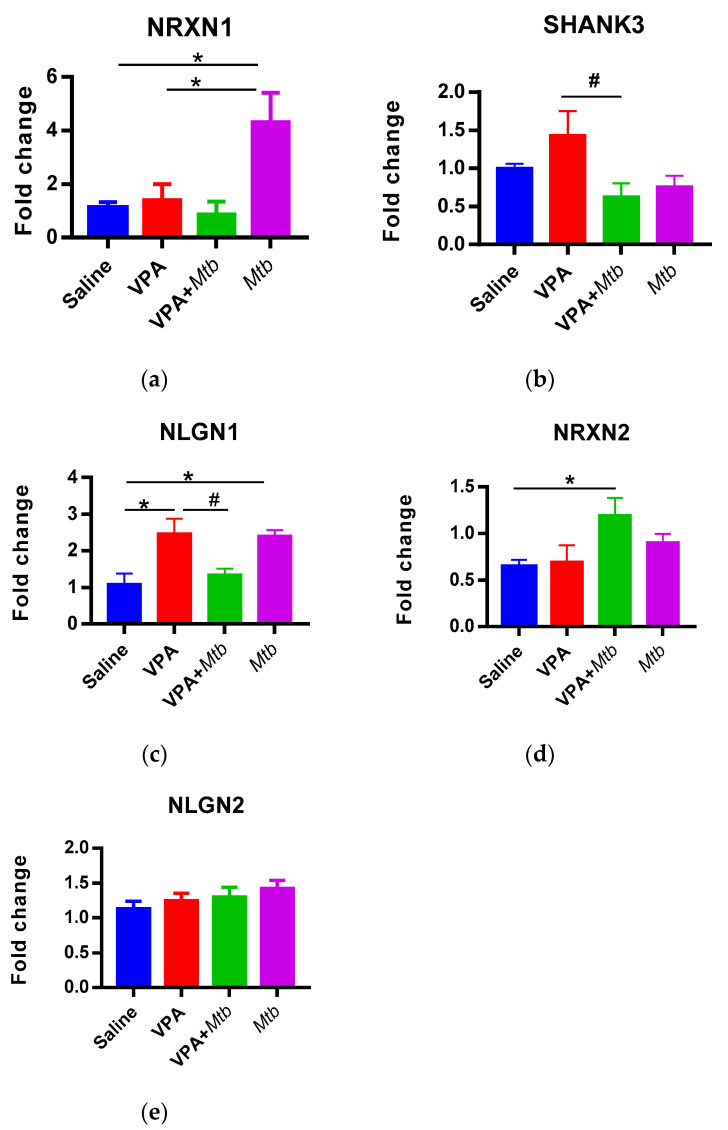
Graphs showing relative fold change in gene expression of (**a**) *NRXN1*, (**b**) NLGN1, (**c**) *NRXN2*, (**d**) *NLGN2* and (**e**) *SHANK3*. *NRXN1* and *NLGN1* are highly expressed in *Mtb* pups (*p* < 0.05) and *NRXN1*, *SHANK3*, *NLGN1* gene expression levels are restored in VPA + *Mtb* offspring. # *p* < 0.05 vs. VPA and * *p* < 0.05 vs. saline, *n* = 5 animals per group (one-way ANOVA). Detailed statistics are available in Appendix B, Table A7.

The expression of *NRXN1*, *SHANK3*, *NLGN1*, *NRXN2* and *NLGN2* genes was measured relative to the *GAPDH* gene (Figure 4). *NRXN1* and *NLGN1* genes were upregulated (*p* < 0.05 vs. saline) in the cerebellum of mice exposed to *Mtb* in utero. Exposure to VPA + *Mtb* upregulated *NRXN2* expression (*p* < 0.05 vs. saline) and downregulated *SHANK3* and *NLGN1* expression (*p* < 0.05 vs. VPA). *Mtb* caused a 2-fold increase in *NRXN1* expression (*p* < 0.05) compared with the positive control (VPA), but it had no effect on the expression of *NRXN2* and *NLGN2* in the cerebellum of infected mice offspring. *Mtb* without VPA significantly upregulated NRXN1 (*p* < 0.001) and NLGN1 (*p* < 0.05) expression compared with VPA + *Mtb* (Appendix B, Table A2).

## 4. Discussion

Peripheral immune dysfunction is a key feature in ASD and MIA offspring. MIA increases the levels of pro-inflammatory cytokines (IL-6, TNF-α and IL-1β) that induce inflammation in the growing fetus; failure of negative feedback control leads to chronic inflammation that persists into the postnatal period. To the best of our knowledge, this is the first report that associates *Mtb* infection with ASD-like phenotype. Our results show an increase in plasma IL-6 and IL-17A following *Mtb*-induced MIA. IL-6 is a key mediator of nervous and immune system cross talk, owing to its ability to cross the blood–brain barrier (BBB). IL-6 levels are elevated in ASD patients [6]. IL-6 is involved in TH17 differentiation, leading to production of IL-17; hence, it is expected that an increase in IL-6 is accompanied by an increase in IL-17A as well. *Mtb* infected mice offspring also showed decreased social skills as revealed by their preference for an object to a mouse (Figure 1b). Furthermore, these animals spent more time with a familiar mouse than an unfamiliar one. Consistent with our findings are impaired social behaviors in Poly I:C-induced MIA offspring [29,30]. Recent findings indicate that IL-17A is a mediator of MIA that reduces social interaction and induces repetitive behaviors in MIA offspring [4,31], suggesting that the elevated plasma IL-17A levels in *Mtb*-induced MIA offspring most probably evoked repetitive behaviors and a lack of social skills in our study. Additionally, elevated plasma IL-6 levels are associated with ASD-like behaviors. Injecting pregnant mice with IL-6 was sufficient to induce ASD-like behaviors, which were rescued by anti-IL-6 antibodies [32]. Taken together, circulating IL-6 and IL-17A somehow influence ASD-like behaviors in *Mtb*-induced MIA offspring.

Furthermore, our results indicate a significant reduction in plasma IFN-γ, IL-1β and TNF-α levels in *Mtb* offspring. This contradicts studies [33,34,35] that reported heightened levels of these pro-inflammatory cytokines in MIA offspring. This could be a result of differing immune response to Poly I:C, LPS and *Mtb*. It is also possible that *Mtb* infection is insufficient to evoke a full spectrum of ASD-associated immune changes that persists into the postnatal life of the offspring. The reduced production of pro-inflammatory cytokines could be an effect of the anti-inflammatory role of IL-6 [36,37] and IL-17A; hence, they can suppress the release of pro-inflammatory cytokines IL-1β and TNF-α [38] while elevating IL-10 to counter inflammation in the immune system. However, there was no change in IL-10 as expected, suggesting a failure of anti-inflammatory regulatory activity. This reduced production in IL-10 is supported by previous reports that did not find a change in IL-10 levels in ASD patients [39,40] and MIA offspring [31].

Finally, we assessed the cerebellar expression profile of genes encoding synaptic molecules that are implicated in ASD pathophysiology. The cerebellum is involved in sociability, emotions and motor coordination; hence, the cerebellum is thought to be involved in developmental disorders characterized by altered social patterns and repetitive behaviors [41]. Our results show that gestational *Mtb* infection does not affect the cerebellar expression of *NLGN2*, *NRXN2* and *SHANK3* but upregulates the expression of *NRXN1* and *NLGN1* genes in offspring. Previous findings indicate that increased expression of *NRXN*s reduces GABAergic neurotransmission [42], while overexpression of NLGN1 increases glutamatergic neurotransmission [43], suggesting an excitation/inhibition imbalance in the synapse that can influence behavior. Increased repetitive behaviors were also observed in other MIA offspring [4,29]. The mechanism underlying repetitive behaviors is not clear; however, they are thought to arise from a combination of genetic and environmental factors affecting the cerebellum development [44,45].

It is interesting and noteworthy that pups born to mothers treated with VPA and *Mtb* (VPA + *Mtb*) did not present systemic inflammation and behavioral deficits; more so, altered cerebellar *NRXN1* expression, social stimulus and self-grooming behaviors were restored to normal levels (saline). Our results are consistent with previous studies in which birth defects were reduced and altered gene expression profiles restored by MIA in teratogen-exposed rodents [46,47]. Although the mechanism behind the rescue mechanism of MIA in rodents exposed to teratogens is not well understood, it has been suggested that MIA can protect against teratogenic effects of VPA through the activity of maternal cytokines that normalize proliferation events and reduce developmental disorders [48,49].

## 5. Conclusions

In conclusion, this study provides new evidence that *Mtb* infection in pregnancy is sufficient to influence brain development such that offspring exhibit increased self-grooming, enhanced systemic inflammation, altered gene expression at synapses and impaired social interaction, which reflect ASD core features. Overall, our study provides new insights and roles of a global pathogen in the MIA pathway implicated in the etiology of ASD. Future studies should focus on measuring maternal cytokines during pregnancy as well as in the brain tissue of *Mtb-*induced MIA offspring.

## Figures and Tables

**Table 1:** Groups and treatments in the study.

Group	Treatment and Dosage	Route of Administration
Saline	0.2 mL of saline	Single i.p.
VPA	0.2 mL of 500 mg/kg valproic acid	Single i.p.
VPA + *Mtb*	0.2 mL of 350 mg/kg valproic acid + 10 mL of 1 × 10^8^ CFU (OD_600_ = 1)	Single i.p. + aerosol infection
*Mtb*	10 mL of 1 × 10^8^ CFU (OD_600_ = 1)	Single aerosol infection

OD—optical density, CFU—colony forming units, i.p.—intraperitoneal.

**Table 2:** Oligonucleotide primer sequences.

Genes	Forward Primer	Reverse Primer	Amplicon Size
*NRXN1*	AGGTTCCGTGTGTCACTTGC	TCCTGTGTGTGTCTGGGGAT	452
*NRXN2*	GCAGGGATTGGACACGCTAT	GAACTGTGACTGCCTACCCC	464
*NLGN1*	GGGGATGAGGTTCCCTATGT	GGTTGGGTTTGGTATGGATG	190
*NLGN2*	TTTCCGTCCTCCCCATCCAAT	TAGGAGCCGCCGTGTAGAAA	923
*SHANK3*	GGCCATTTCAACAGAAGCCC	TGCGCCTTCGATCTCATGG	119
*GAPDH*	CCCTTAAGAGGGATGCTGCC	ACTGTGCCGTTGAATTTGCC	118

## Data Availability

Data will be made available on request.

## Appendix A

**Table A1:** Plasma cytokine concentrations.

Cytokine	*Mtb* Concentration (pg/mL)	VPA + *Mtb* Concentration (pg/mL)
TNF-α	50.7 + 11.21 *	61.9 + 11.21
IFN-γ	19.6 + 4.78 ***	37.7 + 4.78
IL-17A	119 + 10.35 ^a,^#	79.7 + 10.35
Il-10	31.3 + 5.35	34.6 + 5.35
Il-6	7.03 + 0.481 ^b,^**	4.00 + 0.481
Il-1β	3.20 + 0.701 *	2.98 + 0.701 **

^a^*p* < 0.05; ^b^ *p* < 0.0001 vs. VPA + *Mtb*; * *p* < 0.5; ** *p* < 0.001; *** *p* < 0.0001 vs. saline; # *p* < 0.05.

**Table A2:** Gene expression profiles of synaptic molecules.

Gene	*Mtb* Fold Change	VPA + *Mtb* Fold Change
*NRXN1*	4.31 + 0.971 *^,^^a^	0.855 + 0.971 #
*SHANK3*	0.764 + 0.278	0.629 + 0.278 #
*NLGN1*	2.42 + 0.376 *^,b^	1.35 + 0.376
*NRXN2*	0.908 + 0.197	1.19 + 0.197 *
*NLGN2*	1.42 + 0.168	1.30 + 0.168

^b^*p* < 0.05, ^a^ *p* < 0.001 vs. VPA + *Mtb*; * *p* < 0.05 vs. saline, # *p* < 0.05 vs. VPA.

## Appendix B

**Table A3:** ANOVA results for Social stimulus.

ANOVA	DF	F (DFn,DFd)	*p* Value
Interaction	3	F (3, 88) = 14.12	*p* < 0.0001
Row FActor	3	F (3, 88) = 0.04163	*p* = 0.9886
Column Factor	1	F (1, 88) = 13.61	*p* = 0.0004
Residual	88		

**Table A4:** ANOVA results for Social novelty.

ANOVA	DF	F (DFn,DFd)	*p* Value
Interaction	3	F (3, 88) = 7.405	*p* = 0.0002
Row FActor	3	F (3, 88) = 4.065	*p* = 0.0094
Column Factor	1	F (1, 88) = 3.146	*P* = 0,0796

**Table A5:** ANOVA results for Self-grooming.

ANOVA	DF	F (DFn,DFd)	*p* Value
Treatment (between columns)	3	F (3, 44) = 11.29	*p* < 0.0001

**Table A6:** ANOVA results for Cytokines concentration

CYTOKINES	DF	F (DFn,DFd)	*p* Value
TNF-α	3	F (3, 16) = 3.727	*p* = 0.0332
IFN-γ	3	F (3, 16) = 24.97	*p* < 0.0001
IL-17A	3	F (3, 16) = 10.47	*p* = 0.0005
IL-10	3	F (3, 16) = 0.1383	*p* = 0.9356
Il-6	3	F (3, 16) = 15.01	*p* < 0.0001
Il-1β	3	F (3, 16) = 8.232	*p* = 0.0015

**Table A7:** ANOVA results for Gene expression

GENE	DF	F (DFn,DFd)	*p* Value
*NRXN1*	3	F (3, 16) = 5.51	*p* = 0.0086
*SHANK3*	3	F (3, 16) = 3.258	*p* = 0.0492
*NLGN1*	3	F (3, 16) = 7.215	*p* = 0.0028
*NRXN2*	3	F (3, 16) = 3.29	*p* = 0.0478
*NLGN2*	3	F (3, 16) = 1.013	*p* = 0.4127
